# The application of organoids in cancers associated with pathogenic infections

**DOI:** 10.1007/s10238-024-01435-8

**Published:** 2024-07-25

**Authors:** Yuyu Zhang, Tao Liu, Wenting He

**Affiliations:** 1 Department of the Second Clinical Medical College, Lanzhou University, Lanzhou, 730030 China; 2 Second Hospital of Lanzhou University, Lanzhou University, Lanzhou, 730000 China; 3 Gansu Provincial Key Laboratory of Environmental Oncology, Lanzhou, 730030 China; 4 Digestive System Tumor Prevention and Treatment and Translational Medicine Engineering Innovation Center of Lanzhou University, Lanzhou, 730030 China; 5 Digestive System Tumor Translational Medicine Engineering Research Center of Gansu Province, Lanzhou, 730030 China

**Keywords:** Organoids, *H. pylori*, *E. coli*, HBV, HPV, Cancers

## Abstract

Cancers associated with pathogen infections are gradually becoming important threats to human health globally, and it is of great significance to study the mechanisms of pathogen carcinogenesis. Current mechanistic studies rely on animal and two-dimensional (2D) cell culture models, but traditional methods have been proven insufficient for the rapid modeling of diseases caused by new pathogens. Therefore, research focus has shifted to organoid models, which can replicate the structural and genetic characteristics of the target tissues or organs in vitro, providing new platforms for the study of pathogen-induced oncogenic mechanisms. This review summarizes the application of organoid technology in the studies of four pathogen-associated cancers: gastric cancer linked to *Helicobacter pylori*, liver cancer associated with hepatitis B virus or hepatitis C virus, colorectal cancer caused by *Escherichia coli*, and cervical cancer related to human papillomavirus. This review also proposes several limitations of organoid technology to optimize organoid models and advance the treatment of cancer associated with pathogen infections in the future.

## Introduction

In recent years, cancer has emerged as one of the most prevalent diseases, which is seriously harmful to human health and is a leading cause of mortality worldwide. The factors that cause the occurrence and development of cancer are multifaceted, including external physical factors (electric radiation, ultraviolet rays), chemical factors (nitrosamines, aflatoxins), poor lifestyle habits (smoking, drinking, staying up late), genetic factors (gene mutations or deletions), and pathogenic infections. Notably, cancers associated with pathogen infections are also gradually becoming important threats to human health globally, such as gastric cancer linked to *Helicobacter pylori* (*H. pylori*), liver cancer linked to hepatitis B virus (HBV) or hepatitis C virus (HCV), colorectal cancer associated with *Escherichia coli* (*E. coli*), and cervical cancer caused by human papillomavirus (HPV). The global 2022 incidence and mortality rates of gastric, liver, colorectal, and cervical cancers, and prevalence rates of these four cancers over the five-year period from 2018 to 2022 are shown in Table 1(data were obtained from Global Cancer Observatory), which also includes the percentage of these four pathogen-associated cancers in relation to overall cancers.

**Table 1:** Incidence, mortality, prevalence and proportion of pathogen-associated cancers for four cancers

Cancers	Incidence (%)	Mortality (%)	Prevalence (%)	Pathogen-associated cancers/all cancers (%)
Gastric cancer	4.9	6.8	3.0	90.0 [1]
Liver cancer	4.3	7.8	2.2	21.0–55.0 [2]
Colorectal cancer	9.6	9.3	10.8	23.0–67.0 [3]
Cervical cancer	3.3	3.6	3.6	100.0 [4]

**Fig.1:**
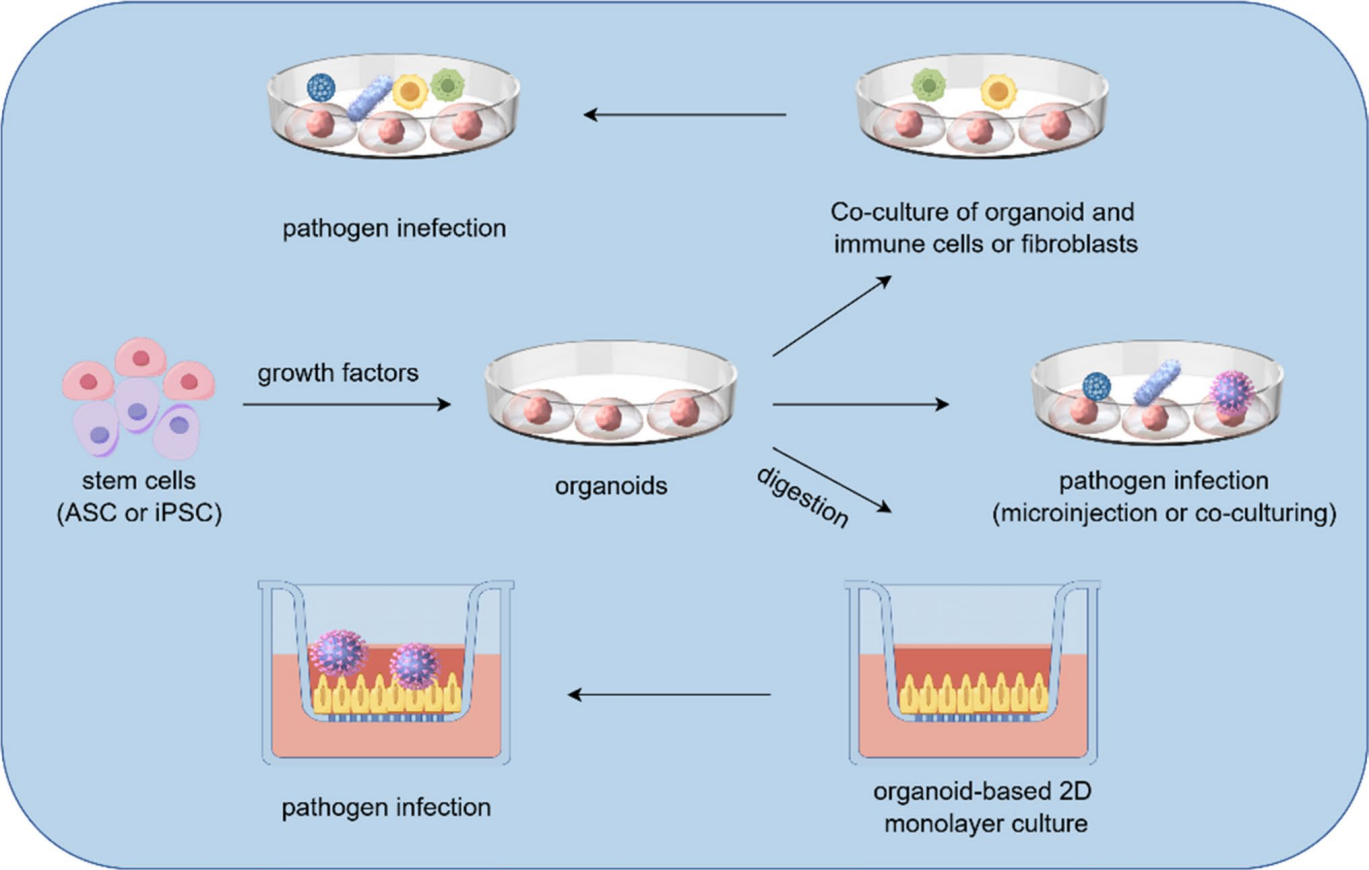
The development and pathogen infection of organoids. Organoids are usually derived from adult stem cells (ASC) or induced pluripotent stem cells (iPSC), replicating the development of targeted tissue and organ in vitro. 3D organoids can be directly infected with pathogens, enzymatically dissociated into 2D monolayers of cells, and reused for the study of pathogen infections. Alternatively, co-cultured with immune cells or fibroblasts and reused for the study of pathogen infection

Classically, pathogen infection studies utilized cancer cell lines or animal models, but both of which have their disadvantages. Standard transformed cancer cell lines often cannot reproduce the essential characteristics of their corresponding cells in vivo counterparts [5]. Similarly, animal models may not always provide a faithful representation of human disease on account of differences between species [5]. Moreover, pathogens typically exhibit a limited species or tissue specificity, infecting only certain species or specific cell types. Consequently, traditional cell lines or animal infection models are increasingly insufficient for pathogen infection research. The lack of functional models that accurately mimic normal human physiology and pathophysiology severely restricts human pathogen infection research. Notably, organoid, 3D tissue models constructed from stem cells through self-assembly in vitro 3D cell culture conditions [6], undoubtedly pave the way for expansive research in this field (Fig. 1).

Typically, organoids are derived from embryonic stem cells (ESC), adult stem cells (ASC) or induced pluripotent stem cells(iPSC), replicating the development of targeted tissue and organ in vitro [7]. Due to ethical constraints associated with the use of ESC, a number of researches concentrate on the utilization of iPSCs and ASCs (Table 2). This includes organoids of stomach, liver, intestines, hepatobiliary, otic, cervix, lung, and hypothalamic arch. This review discusses the application of organoid techniques in exploring the correlation between *H. pylori* and gastric cancer, HBV or HCV and liver cancer, *E. coli* and colorectal cancer, as well as HPV and cervical cancer.

**Table 2:** Organoids generated from induced pluripotent stem cells and adult stem cells

Cell type	Organ or tissue	Reference
Induced pluripotent stem cells	Stomach organoid	[8]
	Liver organoid	[9]
	Intestine organoid	[10]
	Hepatobiliary organoid	[11]
	Otic organoid	[12]
	Hypothalamic arcuate organoid	[13]
Adult stem cells	Cervix organoid	[14]
	Lung organoid	[15]
	Intestine organoid	[16]

### *H. pylori *and gastric cancer

**Fig.2:**
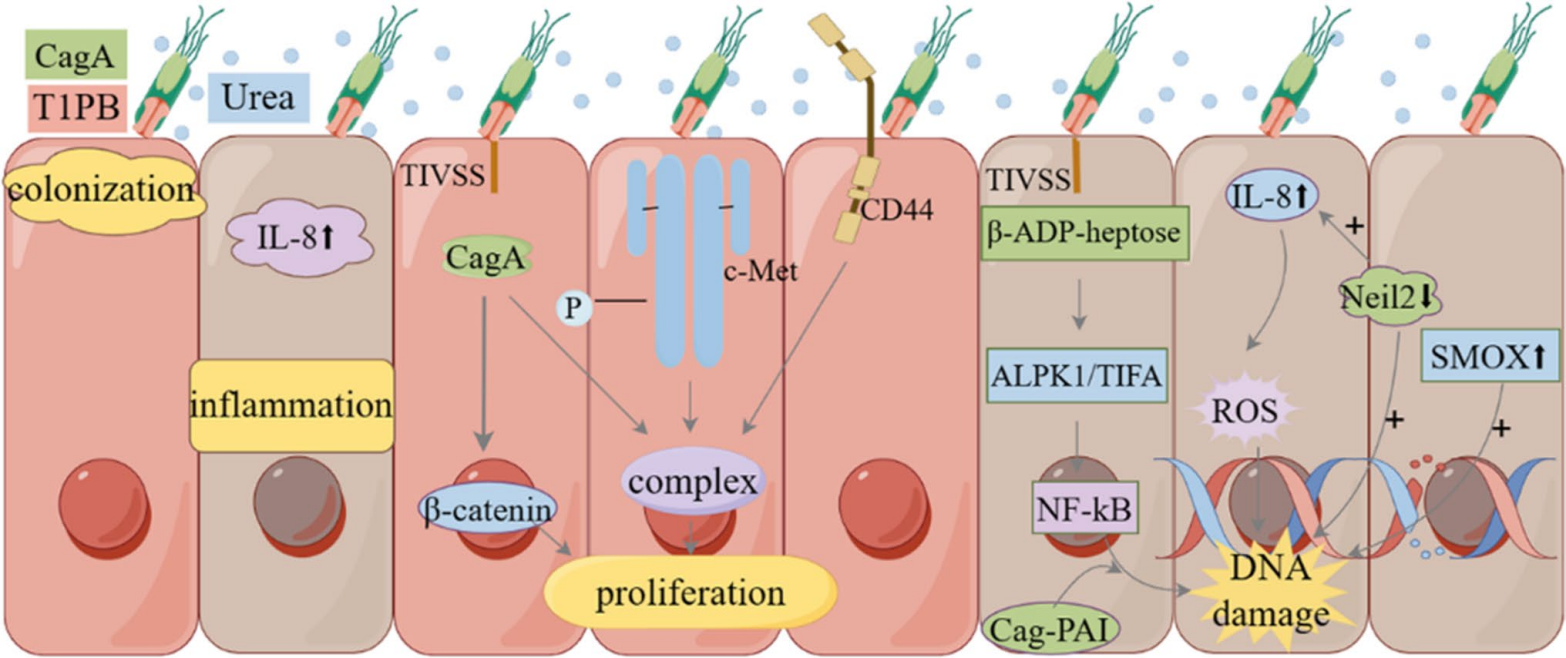
The colonization of the gastric epithelium, induction of the inflammatory response, proliferation of gastric epithelium, and DNA damage caused by *H. pylori*. *H. pylori* senses urea via a chemoreceptor known as *TlpB*, which guides *H. pylori* to the gastric epithelium. *H. pylori* infection triggers the expression of pro-inflammatory cytokines, particularly IL-8. *CagA* translocates via TIVSS system to the cytoplasm of gastric epithelial cells, where it forms a complex with phosphorylated C-Met and CD44, causing proliferation of gastric epithelium. *CagA* causes increased distribution of β-catenin in the nucleus, triggering proliferation of epithelial cells. The ALPK1/TIFA pathway is activated upon TIVSS -system-dependent delivery of β-ADP-heptose into epithelial cells, which in turn activates NF-kB(kappa), thereby causing DNA damage. *H. pylori* infection increases DNA damage by down-regulating Neil2 expression, and the inflammatory response induced by *H. pylori* is Neil2-dependent. Inflammatory cytokines induce ROS production, which in turn triggers DNA damage. *H. pylori* infection increases SMOX expression in human gastric tissues, and SMOX deficiency decreases DNA damage

*H. pylori*, a group of Gram-negative bacilli that bend in a comma, S, spiral, or gull-wing shape, can colonize the stomach or intestines of mammals. Although most carriers of *H. pylori* remain asymptomatic, *H. pylori* infection can result in many gastric disorders such as chronic gastritis, peptic ulcers [17]. Moreover, persistent *H. pylori* infection is considered as a major risk factor for gastric cancer. Although the lifetime risk of gastric cancer in *H. pylori* infected individuals is 1%–5%, approximately 90% of gastric cancer cases are attributed to *H. pylori* infection [1]. Despite considerable knowledge of *H. pylori’s* carcinogenesis, the advent of organoids offers a novel approach to study this process. For instance, McCracken et al. [18] successfully generated human gastric organoids (hGOs) in vitro through the directed differentiation of human pluripotent stem cells. HGOs in this study formed primary gastric glands and multiple gastric endocrine cells [18]. The study also successfully demonstrated that hGOs could be used to mimic the pathophysiological responses of the human stomach to *H. pylori*, suggesting that organoid models can be effectively utilized to study *H. pylori* carcinogenesis. And in another study, Hiroshi Ota et al. [19] succeeded in establishing a co-culture model in which immune cells maintain activity and keep contact with hGOs. This allowed for real time observation of immune responses and tumor growth over time, effectively replicating immune responses in vivo [19]. Although organoids may not fully replicate a state of chronic inflammation spanning decades, they have been instrumental in studying various mechanisms of cancer progression [20]. This paper summaries key findings that highlight the advantages of organoids, including colonization of the gastric epithelium, induction of the inflammatory response, proliferation of gastric epithelium, and DNA damage caused by *H. pylori* (Fig. 2).

### Colonization of *H. pylori*

The colonization of the gastric epithelium by *H. pylori* is the initial step in the onset of gastric cancer. Huang et al. [21] utilized epithelial secretions from uninfected hGOs as a conditioned medium to study this process. Their research revealed that *H. pylori* senses urea via a chemoreceptor known as *TlpB*, which guides *H. pylori* to the gastric epithelium [21]. Another study on *H. pylori* colonization, reported that its presence at the site of injury impedes the repair process in the mouse stomach. The high-intensity light (two-photon photodamage, PD) was employed to produce cellular injury on mouse gastric organoids and used confocal microscopy to monitor the injury site [22]. Subsequently, these organoids were infected with wild-type (WT) *H. pylori* and *H. pylori* mutants that were non-motile (*ΔmotB*), non-chemotropic (*ΔcheY*), and lacking a single chemoreceptor (*ΔtlpA, ΔtlpB, ΔtlpC, or ΔtlpD*). It was found that none of the mutants lacking *motB*, *cheY*, and *tlpB* could accumulate at the injury site post PD [22]. In contrast, WT *H. pylori* and mutants lacking *tlpA, tlpC*, and *tlpD* congregated at the injury site within 5 min post PD, thereby inhibiting epithelial recovery. These findings suggest that both bacterial motility and chemotactic signaling are crucial for *H. pylori* accumulation at the site of injury, with *TlpB* serving as the chemoreceptor that enables *H. pylori* to detect the injury site [22].

### Inflammatory response induced by *H. pylori*

*H. pylori* infection triggers inflammatory responses, with the onset of inflammation implicates the expression of pro-inflammatory cytokines, particularly IL-8 [23]. Uotani et al. [23] utilized hGOs-transformed monolayer primary gastric epithelial cells (gastroid monolayers) to examine IL-8 expression induced by *H. pylori* infection. The results showed that IL-8 levels in hGOs were significantly increased in the *H. pylori* infected group compared to the uninfected group. However, IL-8 expression levels were comparable between gastroid monolayers infected with WT *H. pylori* and mutant *H. pylori* strain lacking *Cag PAI* (*ΔCag PAI*) [23].This suggests that the increase in IL-8 expression resulting from *H. pylori* infection is not related to *Cag PAI*. *H. pylori* infection also secretes the pro-inflammatory cytokine TNFα. To clarify the role of secreted TNFα, Kenly Wuputra et al. [24] added prepared TNFα recombinant protein to cultures of *H. pylori* microinjected into the lumen of human gastric cancer-derived organoids. The results showed that, at a high concentration of 500 ng/mL TNFα, all the *H. pylori* infected organoids showed reduced cell viability and inhibited invasive activity [24]. It suggests that *H. pylori* infection secretes TNFα and promotes inflammatory response.

### *H. pylori* -induced proliferation of gastric epithelium

*H. pylori* infection disrupts homeostasis and induces proliferation of the gastric epithelium. An important discovery was revealed that β-catenin plays a crucial role in the proliferation induced by *H. pylori* infection [25]. In this study, mice were pretreated with Cardionogen 1, an inhibitor of β-catenin, in gastric organoids before being infected with *H. pylori*. The proliferative capacity of the infected gastric organoids was significantly reduced following pretreatment with Cardionogen 1 [25].

McCracken et al. showed that proliferation of gastric epithelial induced by *H. pylori* infection is mediated by c-Met and regulated by *CagA*. To simulate a typical host–pathogen interface and to assess the signaling and proliferation of the gastric epithelium, both pathogenic and attenuated *H. pylori* were microinjected into the epithelial lumen of hGOs [18]. These hGOs were derived from directed differentiation of human pluripotent stem cells. During 24 h, it was noticed that both types of bacteria were closely associated with the hGOs’s epithelium. Additionally, the phosphorylation of c-Met and the proliferation of epithelial cell were also observed. Furthermore, when hGOs were injected with *H. pylori* strains expressing a deletion of *CagA* (*ΔCagA*), the proliferative response was absent. In contrast, *CagA* from WT *H. pylori* translocated to the epithelial cells of hGOs and rapidly bound to the c-Met receptor, forming a complex [18]. This complex activated signaling, thereby inducing epithelial proliferation. Bertaux-Skeirik et al. [26] reached a similar conclusion in another study. Using mouse gastric organoid as a research model to detect the proliferative changes in gastric epithelial cells induced by *H. pylori* infection. It was found that the proliferation of epithelial cells in *H. pylori* infected mouse gastric organoids was significantly increased compared to the uninfected group. However, the *H. pylori*-induced proliferative response was remarkably inhibited when pretreated with a c-Met inhibitor in the organoids [26]. Interestingly, hGOs infected by *ΔCagA* of *H. pylori* did not exhibit a difference in proliferation compared to the uninfected group. Furthermore, gastric organoids from CD44-deficient (*CD44KO*) mice show no proliferative response to the infection of *H. pylori *[26], unlike those from infected WT mice. These suggests that *H. pylori*-infected proliferation in gastric epithelial cells were mediated by activation of c-Met signaling. While, *CagA* and β-catenin may co-regulate this proliferation, CD44 appears to play a functional role in the process.

### DNA damage induced by *H. pylori*

Given that *H. pylori* infection typically progress into a chronic infection, the associated sustained inflammatory response and reactive oxygen species (ROS), to the contribute to the accumulation of DNA damage, thereby facilitating the development of gastric cancer [27]. In their studies on human-derived organoids, Bauer et al. [28] reported that *H. pylori* infection induces DNA damage in S-phase cells in an ALPK1/TIFA/NF–kB(kappa)-dependent manner. The IL-8 response and DNA damage induced by infecting gastroid monolayers with WT *H. pylori* were found to be similar to those, induced by exposure to *H. pylori* LPS biosynthesis intermediate (β-ADP-heptose). However, this DNA damage was restricted to S-phase cells. Infection with WT *H. pylori* resulted in higher levels of both DNA damage and IL-8 expression than infection with *H. pylori* mutants lacking the *rfaE* enzyme responsible for β-ADP-heptose production or *ΔCag PAI *[28]. This suggests that the S-phase specific DNA damage induced by *H. pylori* is dependent on the bacterium’s *rfaE* enzyme and *Cag PAI*, and can be induced by adding the ALPK1 ligand β-ADP-heptose [28]. Additionally, Neil2 and spermine oxidase (SMOX) have been associated with DNA damage induced by *H. pylori* infection. Sayed et al. [27] conducted an experiment, where they infected enteroid-derived monolayers (EDMs) from the stomachs of both WT and Neil2 knockout (*Neil2KO*) mice, as well as humans, with *H. pylori*. They discovered that *H. pylori* infection significantly reduced the expression of Neil2. Inflammatory cytokines (such as IL-8, IL-6, and MCP-1) were highly expressed in EDMs from both mice and human, and their expression was further up-regulated in *Neil2KO* mice EDMs. Whereas, *Neil2KO* mice EDMs exhibited a higher accumulation of oxidative DNA damage compared with WT mice [27]. These findings suggest that *H. pylori* infection, by reducing Neil2 expression, increases the accumulation of oxidative DNA damage. Furthermore, the inflammatory response in stomach epithelial cells infected by *H. pylori* appears to be Neil2-dependent [27]. In another experiment, DNA damage and β-catenin activation were found to be reduced in gastric organoids of *H. pylori*-infected the mice lacking Smox, compared to gastric organoids of *H. pylori*-infected WT mice [29]. The role of SMOX in mediating *H. pylori*-induced β-catenin activation was demonstrated in hGOs treated with a second-generation SMOX inhibitor [29]. These findings suggest that SMOX may contribute to the oncogenic effects of *H. pylori* infection by inducing DNA damage and the activation of β-catenin signaling [29]. And in *H. pylori* carcinogenesis, CDK1 could act as a signaling bridge that connects infection, inflammatory signaling, and β-catenin activation, based on studies in mouse gastric organoids [30].

## HBV, HCV and liver cancer

**Fig.3:**
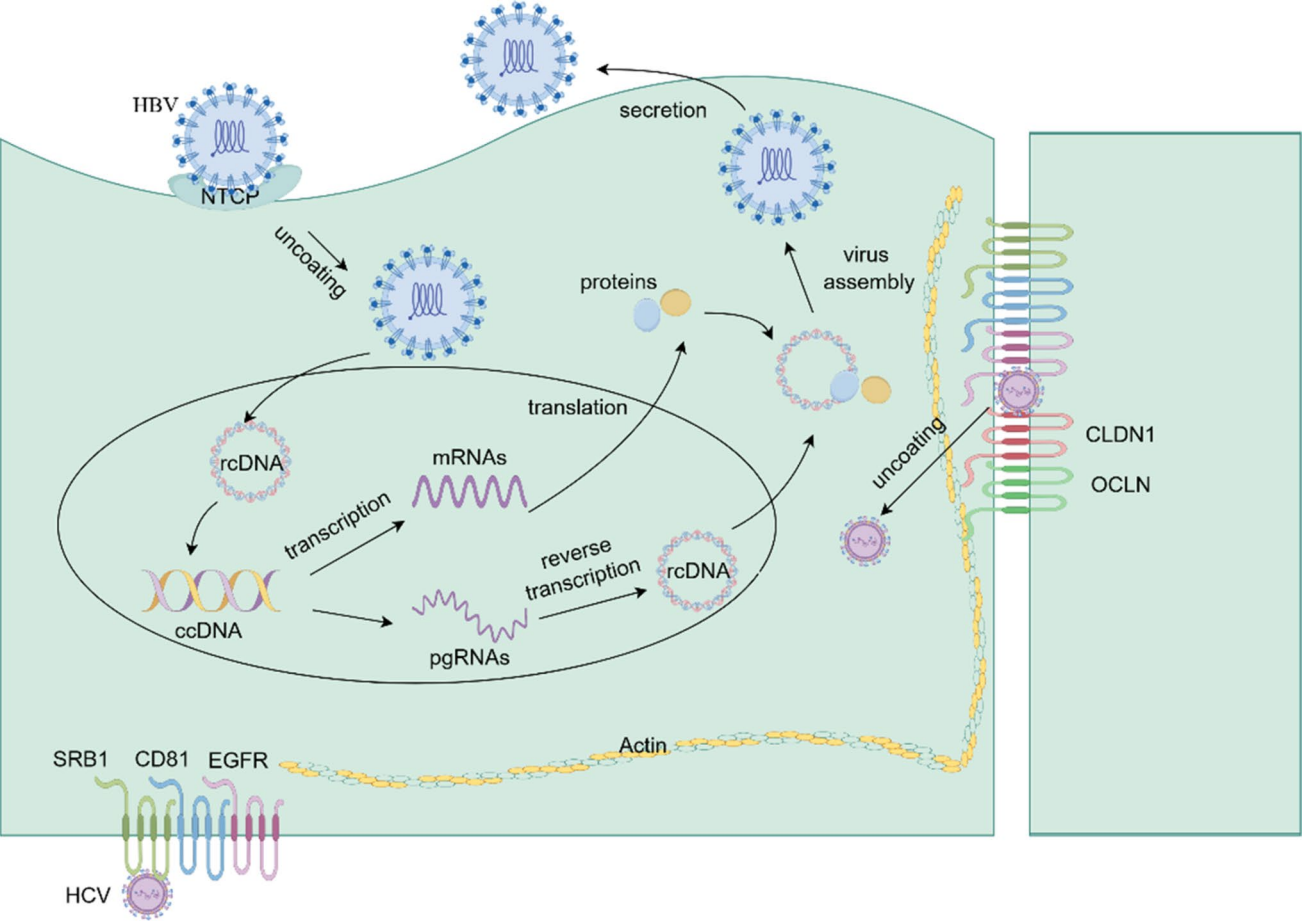
HBV life cycle and HCV entry into the hepatocyte. HBV interacts with the NTCP receptor, followed by viral uncoating and entry into the cell. HBV releases its double-stranded, relaxed-circle DNA (rcDNA) into the nucleus, where it is converted into covalently closed-circle DNA (cccDNA). cccDNA serves as a transcriptional template for the pre-genomic RNA (pgRNA) and the rest of the mRNAs are transcriptional templates. pgRNA is reverse transcribed into rcDNA in the nucleus, and the remaining mRNAs are translated into the corresponding proteins. Prepared proteins and rcDNA are assembled into HBV viruses and released from the cell

Both HBV and HCV are important risk factors for liver cancer. Primary liver cancers are predominantly hepatocellular carcinomas (HCC) (75%–85%) and intrahepatic cholangiocarcinoma (ICC) (10%–15%), and chronic infection with HBV or HCV accounts for 21%–55% of HCC worldwide [2]. HBV, the smallest known DNA virus capable of infecting humans, belongs to the Hepadnaviridae. The HBV genome is an incompletely closed loop double-stranded DNA, with two strands of varying lengths. The shorter strand is the positive strand, while the longer strand is the negative strand. The latter has a fixed length, approximately 3.2 kilobase pairs (kb) and is covalently linked to the viral polymerase at the 5′ end. HBV infects hepatocytes by binding to the host cell receptor sodium taurocholate cotransporter polypeptide (NTCP) through the pre-S1 structural domain of hepatitis B virus surface antigen (HBsAg) [31]. This process is followed by hepatocytes entry through the fusion and internalization of membranes (Fig. 3).

HCV belongs to the Hepacivirus genus of the Flaviviridae family, and is spherical, enveloped, and has a single positive-stranded RNA core. Both HBV and HCV can cause chronic infection and promote liver cancer. Previously, the primary models employed to investigate HBV carcinogenesis were hepatocellular carcinoma cell lines, animal models and primary human hepatocytes (PHH). However, these models all present limitations. Hepatocellular carcinoma cell lines are not suitable for studying tumorigenesis resulting from HBV infection due to their inherent tumor-organ gene expression profile. Animal models, such as mice, are challenging to utilize for studying natural HBV infection due to virus’s strict host and cytophilic nature [31]. PHH were previously regarded as the gold standard for HBV studies, but they are hard to acquire and maintain [31]. Therefore, the exact molecular mechanisms of HBV replication and tumorigenesis remain elusive because of inapposite model systems. And organoid technology offers a promising solution, with its ability to maintain primary 3D cell culture systems that retain the original tissues and functions. Nie et al. [32] utilized human iPSC to culture a functional liver organoid (LO) that retained the donor’s genetic context. This organoid effectively replicated the host-virus interactions by mimicking the HBV viral life cycle, and demonstrated HBV-induced liver function abnormalities. The results suggest that this LO is a reliable and feasible personalized infection model for individualized liver cancer research and treatment. Currently, LOs are being used to study the mediators of HBV entry into hepatocytes and the cellular origin of HBV-associated ICC. Crignis et al. [33] proposed that overexpressing NTCP in LOs through lentiviral transduction does not enhance their susceptibility to HBV. In their studies, LOs were cultured in both expansion medium (EM) and differentiation medium (DM), and then infected with HBV. The results indicated a higher level of HBV infection and replication in organoids cultured in DM compared to those in EM organoids, which was consistent with the higher NTCP expression in DM organoids [33]. The application of the NTCP inhibitor resulted in a reduction in infection levels within the organoids, as evidenced by a decrease in intracellular HBV RNA, HBV DNA and HBV early antigen (HBeAg) in the supernatant of the infected organoids [33]. This suggests that HBV infection in LOs is NTCP-dependent. However, the lentiviral transduction of NTCP overexpression in organoids did not result in an increase in HBV DNA and HBsAg levels in the supernatants of parental organoids. This implies that NTCP expression alone is insufficient to enhance HBV infectivity in liver organoids grown in EM, and additional host factors are required for efficient HBV infection [33]. In another study, Song et al. [34] demonstrated that HBV-associated ICC may originate from hepatocytes. They used an ICC tissue-derived organoid model, generating LOs from freshly collected ICC tissues and appropriate adjoining tissues. The results showed that HBsAg was expressed only in hepatocytes of normal tissues, not in intrahepatic bile duct epithelial cells [34]. This suggests that normal bile duct epithelial cells have no HBV infection and that HBV-associated ICC may originate from hepatocytes [34].

Currently, LOs have also been applied to study the oncogenic process of HCV. This includes the process of HCV entering hepatocytes (Fig. 3), the transcriptional reprogramming of stem cells induced by HCV infection, and co-culture of immune cells with LOs derived from HCV patients. Baktash et al. [35] fluorescently labeled HCV particles with the lipophilic dye DiD, and the HCV entry process was determined by imaging infection of hepatocellular cancer organoid with fluorescent HCV virus particles. DiD-HCV particles were associated with the early entry factor, such as differentiation 81 (CD81), scavenger receptor class BI (SRB1), and epidermal growth factor receptor (EGFR), which were co-localized in the basolateral membrane, and over time, these fluorescent HCV particles migrated in an actin-dependent way at tight junctions; at tight junctions, HCV encounters the late receptor claudin-1 (CLDN1) [35].The interaction between CD81 and CLDN1 localizes the HCV receptor complex near the occludin (OCLN) and internalizes HCV via the EGFR. HCV entry can lead to chronic inflammatory necrosis and fibrosis of the liver, which may progress to cirrhosis and even HCC in some patients. Despite the effective antiviral drugs that can cure HCV, they are unable to reverse end-stage liver disease. Therefore, LOs derived from adult stem cells can be utilized to understand how HCV infection leads to the progression of end-stage liver disease and also assist in the treatment of end-stage liver disease. The presence of HCV RNA was detected in approximately 20% of the cells in LOs obtained from ASC of HCV-infected HCC patients, and the maintenance of low-grade infection by HCV in LOs was first demonstrated by Nathan L.Meyers et al. [36]. Single-cell transcriptional profiling of HCV-infected organoid demonstrated that HCV infection affects stem cell differentiation through influencing the expression of differentiation genes, upregulating the expression of the tumor stem cell marker OCT4, causing transcriptional reprogramming, interfering with stem cell differentiation, promoting tumorigenesis, and disrupting stem cell proliferation and interferon signaling [36]. This may hinder the ability of hepatic stem cells to regenerate liver tissue and increase the possibility of liver injury in patients with chronic infections. And it has been studied that in the presence of patient-derived HCV non-structural protein 3 (NS3) specific peptide KLVALGINAV-specific CD8 + T cells targeted to kill HCV-infected LOs, resulting in 80% of the organoids being considered nonviable at 60 h [37]. In this study, Vaishaali Natarajan et al. [37] developed a microfluidic co-culture system of CD8 + T cells and LOs derived from the ASC of HCV patients with a static microfluidic chip to graphically simulate the physiological interactions between solid tissues, liver and circulating T cells.

## *E. coli* and colorectal cancer

**Fig.4:**
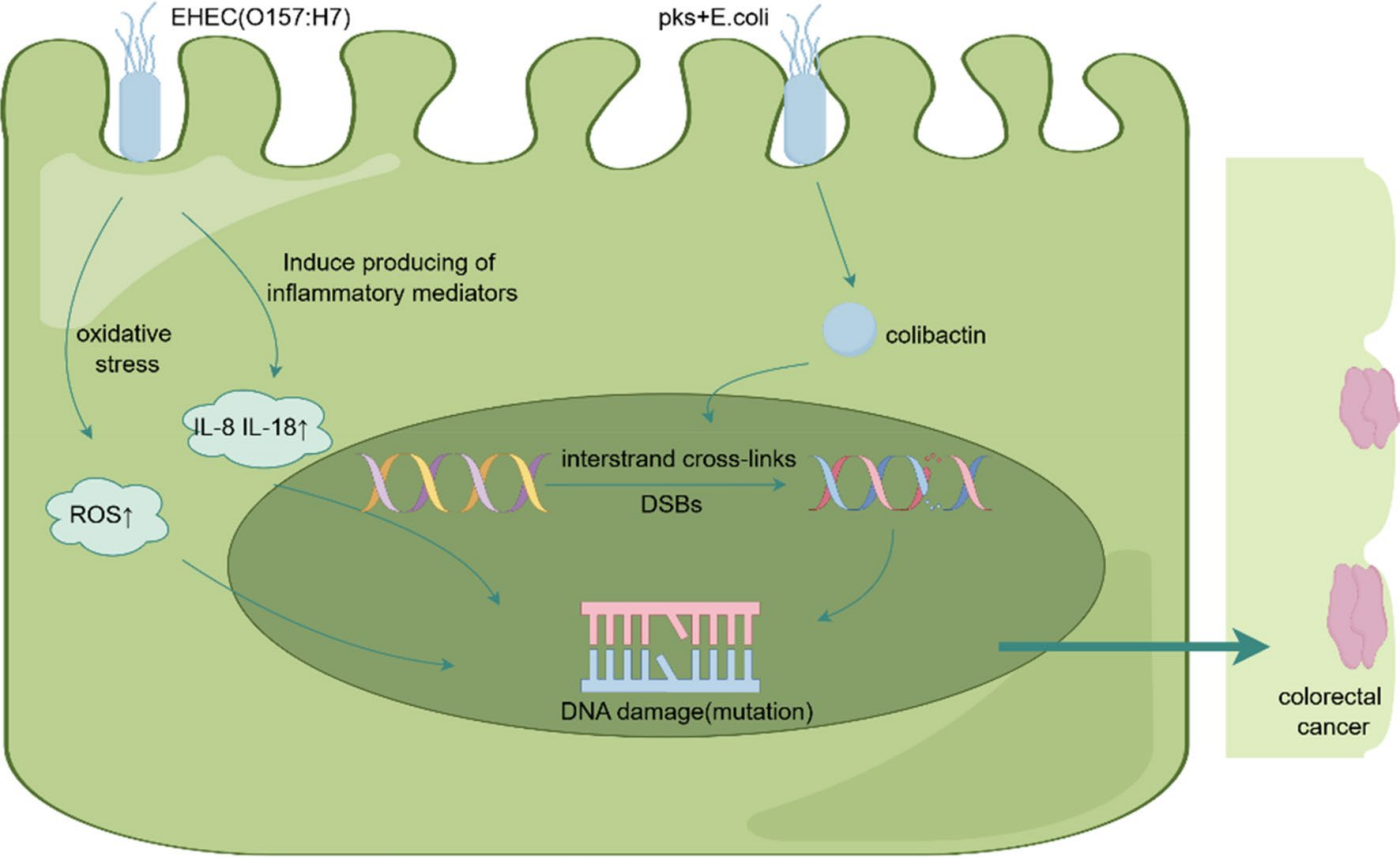
EHEC O157:H7 infection causes oxidative stress ROS production, and induces an increase in the expression of inflammatory mediators IL-8 and IL-18, followed by DNA damage; pks + *E. coli* infection of intestinal cells releases colistin, which triggers DNA double-strand breaks and interstrand cross-links, leading to DNA mutations and ultimately colorectal cancer

*E. coli*, a flagellated, motile, non-budding, Gram-negative bacillus, is a member of the bacterial family of Enterobacteriaceae. It is the most frequent commensal bacteria of the human gastrointestinal tract and is also a significant pathogen. As a symbiotic bacterium, *E. coli* typically exits in a mutually beneficial relationship with its host, seldom causing diseases [38]. However, pathogenic enterogenic *E. coli* strains, predominantly pks + *E. coli*, are also present in the intestinal tract, and pks + *E. coli* have been isolated from approximately 23%–67% of colorectal cancer patients [3]. Interestingly, the development of organoid models has facilitated the study of the mechanisms through which pathogenic *E. coli* initiates colorectal cancer. For example, Pleguezuelos-Manzano et al. [39] confirmed that pks + *E. coli* induces DNA damage in the organoid model by pks + *E. coli* strain co-culturing with human intestinal organoids. This suggests that organoid models are effective tools for studying the carcinogenesis of pathogenic *E. coli*. This paper will summarize the advancements in research using human intestinal organoids (HIOs) to study the association of pathogenic *E. coli* with colorectal cancer, including the response to infection by EnteroHaemorrhagic *E. coli* (EHEC) O157:H7, the DNA damage induced by *E. coli* carrying the pathogenicity island pks (pks + *E. coli*) and its transformation potential in vitro (Fig. 4).

The response to *E. coli* infection in the intestinal tract was simulated by microinjecting pathogenic EHEC O157:H7 and non-pathogenic commensal *E. coli* into the lumen of HIOs [40]. The results showed a time-dependent damage to HIOs by O157:H7, which disrupted the epithelial barrier of HIOs. In comparison to HIOs infected with commensal *E. coli*, those infection with O157:H7 exhibited a remarkable increase of ROS, the inflammatory mediators IL-8 and IL-18, and a significant decrease of the NOD-like receptor NLRC4 [40]. The inflammatory mediator IL-8 was found to be associated with neutrophil recruitment. Further studies revealed significant neutrophil recruitment at the margins of HIOs infected with O157:H7, as compared to commensal *E. coli*, with neutrophils migrating and localizing in the lumen. In conclusion, this study suggests a time-dependent impairment of HIOs by infection with pathogenic O157:H7 strains, which also triggers innate defenses, including ROS production and some inflammatory immune responses [40]. Wilson et al. proposed that colibactin can produce the mutagenic effects through DNA alkylation and triggering double-strand breaks (DSBs) [41]. In another study, Pleguezuelos-Manzano et al. investigated DNA damage induced by pks + *E. coli*, an *E. coli* implicated in a colorectal cancer development. They microinjected pks + *E. coli*, derived from biopsy, into the lumens of HIOs. The mutagenic effect of pks + *E. coli* was confirmed by the observation that HIOs infected by pks + *E. coli* displaying DSBs and interstrand cross-links, compared with the HIOs injected with a homozygous negative control strain that was unable to produce colibactin [42].However this experiment did not explore the transforming potential of colibactin. In another study, Iftekhar et al. [43] investigated the transformative potential of colibactin through the short-term exposure of primary mouse colonic epithelial cell-derived organoids to pks + *E. coli*. They discovered that organoids briefly infected with pks + *E. coli* could generate mutant organoids that were Wnt-independent for growth in Wnt-free medium. Moreover, the Wnt-independent colon organoids generated through the action of colibactin, demonstrated the higher capacity of organoid-forming and expansion in culture [43]. These organoids also showed a significant enrichment of Lgr5 characterized genes, an increased expression of Wnt target genes, and a loss of differentiation markers. Consequently, infection-induced organoid Wnt independence is associated with an impaired cell proliferation phenotype and differentiation [43].

## HPV and cervical cancer

**Fig.5:**
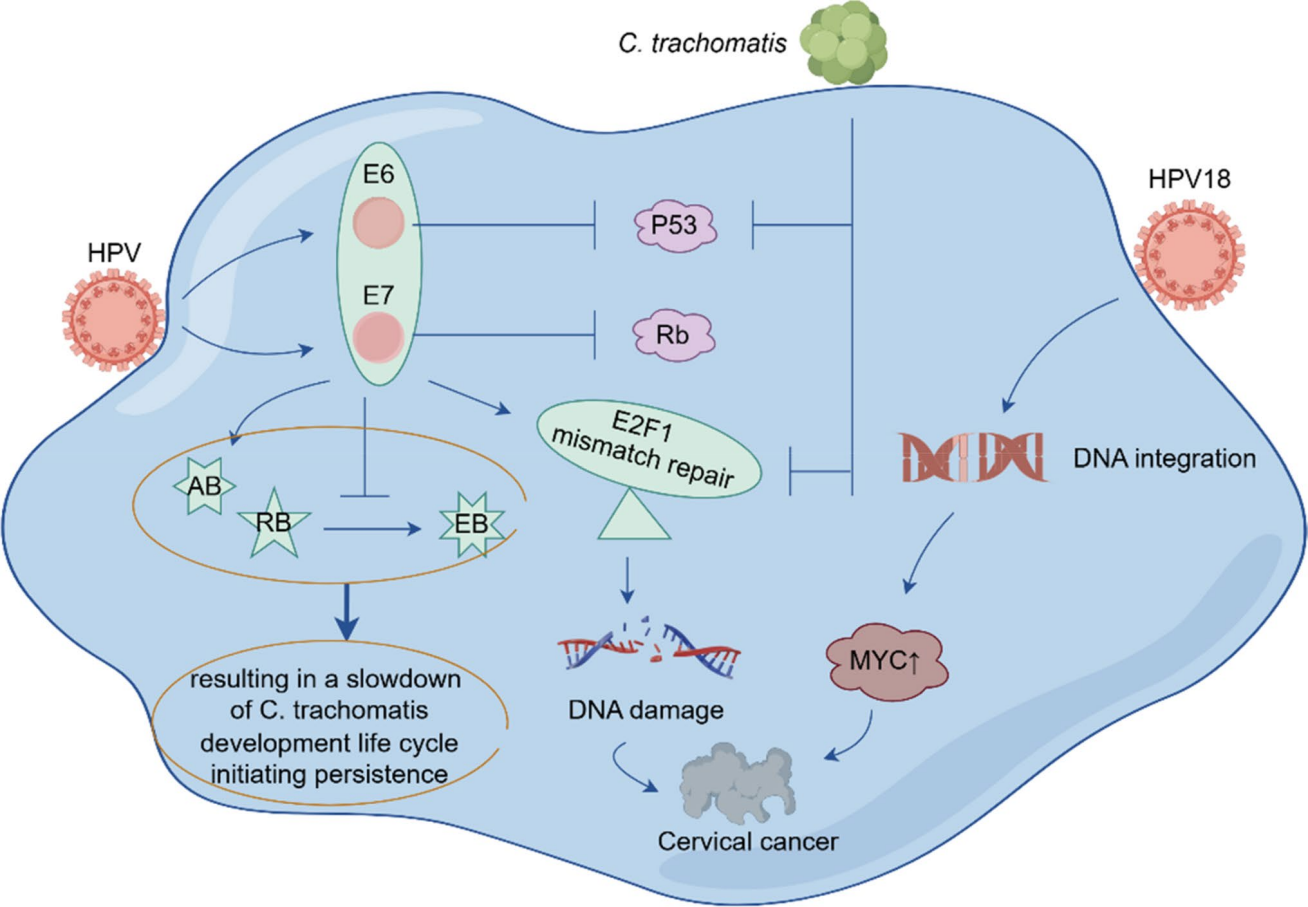
HPV16 E6E7 viral oncoproteins inhibit P53 and R6 tumor suppressor proteins, respectively; HPV infection significantly up-regulates E2F1 target genes; whereas *C. trachomatis* infection also leads to the degradation of P53 proteins but down-regulation of E2F1 target genes; during co-infection of HPV with *C. trachomatis*, *C. trachomatis* overrides HPV down-regulated E2F1 target genes that causes DNA damage; in co-infection, HPV E6 and E7 increase the number of aberrant chlamydial developmental forms (ABs), while inhibiting the redifferentiation of noninfectious replicative reticulate bodies (RBs) to non-replicative elementary bodies (EBs), leading to a slowing of the developmental life cycle of *C. trachomatis*, which initiates persistent infection

HPV is a circular DNA virus with a double-stranded, closed loop genome of approximately 8 kb and an envelope-less, icosahedral capsid. Over 100 genotypes of HPV exist, with HPV 16/18 being the most aggressive and most likely to persist [44]. These genotypes integrate with the host genome and contribute to cervical tumorigenesis. Cervical cancer is the first cancer thought to be eliminated through prevention, as HPV infection is the necessary cause of all cervical cancers [4]. Therefore, it is crucial to continue in-depth research on the mechanism of HPV carcinogenesis. Research has established that the primary region for HPV infection and ensuing cancer progression is the metaplastic epithelium of the transformed zone (TZ) [45]. In one study, Maru et al. [45] successfully cultured organoids originated from the native squamocolumnar junction (SCJ) region and metaplastic squamous cells in the TZ utilizing 3D culture approaches. These organoids retained many SCJ characteristics, effectively imitating cervical SCJ cells. This provides a new approach to study the development of cervical cancer caused by HPV. And the cervical organoids in vitro derived from healthy cervical tissues were established [46].These organoids could be maintained stably over long-term passaging, and were capable of recapitulating disease phenotypes. In another study conducted by Bai Hu et al. [47], cervical cancer organoids were co-cultured with HPV-associated antigenic peptide-activated peripheral blood immune cells from a healthy donor. The results showed that the immune cells disrupted the structure of carcinoid. This suggests that organoids could provide valuable insight into the pathogenesis of cervical cancer. Currently, cervical organoids are being used to study the integration of HPV18 DNA into the host cell genome and the impact of HPV co-infection with *Chlamydia trachomatis* (*C. trachomatis*) (Fig. 5).

In HPV- associated cancer, HPV DNA incorporation produces a super- enhancer- like component to actuate high levels of oncogene expression [48]. Kusakabe et al. [49] proposed that HPV18 integration increases the expression of the proto-oncogene MYC. In this study, Kusakabe et al. established organoids based on cervical SCJ cells from a biopsy sample of a patient who was diagnosed with HPV18-positive small cell carcinoma of the uterine cervix (SCCC, a rare and highly malignant HPV-associated cancer) [49]. RNA-seq analysis of the organoids showed HPV18 was integrated into chromosome 8 at 8q24.21, leading to up-regulation of the expression of the proto-oncogene MYC [49]. This suggests that HPV18 integration into chromosome 8 triggers DNA modifications in the host cell genes, thereby increasing the expression of the host cell proto-oncogene MYC, and potentially inducing the development of cervical cancer. Studies indicates that, while more than 80% of women will contract HPV in their lifetime, under 2% of women will suffer cervical cancer [50]. Typically, patients with cervical cancer are co-infected with both HPV and *C. trachomatis.* Scientists hypothesize that this co-infection is an important factor in the formation of malignant tissues, although the precise mechanisms remain unclear. In a study, Koster et al. [50] successfully created a cervical cancer organoid model that simulates co-infection with HPV and *C. trachomatis*. Their fundings revealed that this co-infection triggers unique cellular reprogramming in the host, with multiple genes being up-regulated or down-regulated in different ways [50]. This impacts the host’s immune response and DNA damage repair mechanism. This study further reveals that co-infection with HPV E6E7 and *C. trachomatis* detrimentally affects both cellular and genomic stability, thereby promoting the progression of cervical cancer. *C. trachomatis* has also been identified as a synergistic factor in HPV carcinogenesis.

## Conclusion

In summary, organoids effectively replicate host infections, encapsulate key features of diseases, and reveal host–pathogen interactions. Compared to traditional approaches such as transformed cell lines or animal models, organoids more accurately mimic the genomic and histological structure of original tissues. They yield faster results faster and predict human responses more accurately in vivo, and they can also be co-cultured with immune cells to demonstrate the immune response in vivo. Moreover, the issue of organoids vascularization has been solved, enhancing their reliability as a model for studying pathogen-induced carcinogenesis [51, 52]. Despite these unique advantages, organoids do have certain limitations and shortcomings in vitro modeling. Firstly, in most situations, organoid approaches to studying pathogen infections are limited to epithelial innate responses at the organ level and do not mimic interorgan communication [53]. Secondly, many researches using organoids to study pathogen infections are limited to single classes of pathogens and lack a microbial community component [53]. Thirdly, however, some organoid models lose certain specific cellular components in culture, which may affect the formation of the organoid lumen and thus the microinjection of pathogens. Finally, short-term survival of organoids in vitro does not adequately demonstrate the long-term effects associated with cancer development.

This review summaries the application of organoids in the studies of four pathogen-associated cancers: gastric cancer caused by *H. pylori*, colorectal cancer related to *E. coli*, liver cancer linked to HBV and HCV, cervical cancer associated with HPV. However, many pathogen-related tumors have not yet been studied using organoid models, such as vaginal cancer, nasopharyngeal carcinoma, Kaposi’s sarcoma, and so on. Nevertheless, we believe that with the rapid development of organoid technology and the continuous improvement of organoid culture models, organoids will undoubtedly play an invaluable role in human disease research and tissue regeneration medicine.

## Data Availability

Data sharing not applicable to this article as no datasets were generated or analyzed during the current study.
